# Validated ligand geometries for macromolecular refinement restraints and molecular-mechanics force fields

**DOI:** 10.1107/S2059798326000975

**Published:** 2026-02-18

**Authors:** Nigel W. Moriarty, David A. Case, Dorothee Liebschner, Paul D. Adams

**Affiliations:** a Molecular Biosciences and Integrated Bioimaging Division Lawrence Berkeley National Laboratory Berkeley CA94720 USA; b Department of Chemistry and Chemical Biology, Rutgers University, Piscataway, NJ08854, USA; c Department of Bioengineering University of California, Berkeley Berkeley CA94720 USA; Global Phasing Ltd, United Kingdom

**Keywords:** macromolecular crystallography, ligand restraints, refinement, *Amber*, molecular mechanics, macromolecular refinement

## Abstract

In macromolecular structure refinement, the low observation-to-parameter ratio and the lack of high-resolution data are countered by using *a priori* information in the form of restraints. Having accurate geometries of the chemical entities in the sample is paramount for generating accurate chemical restraints and, therefore, accurate macromolecular structures. In particular, it is desirable to have accurate restraints for known and novel ligand entities. Quantum mechanics (QM) can minimize the energy of a ligand by adjusting its geometry, and these geometries can be used to generate restraints for macromolecular refinement. This article describes a library of approximately 37 000 small molecules extracted from the Chemical Component Dictionary in the Protein Data Bank and minimized by density-functional QM. The library includes restraint files for use in crystallography or cryo-EM refinement, along with files suitable for molecular-dynamics simulation. Because the geometries are validated using the Cambridge Structural Database, the restraints library provides users with both functional restraints and minimized geometries. This work also provides procedures for generating new and accurate restraints.

## Abstract 2

A database of validated ligand restraints computed using various levels of quantum mechanics is generated and distributed with *Phenix* to streamline and improve macromolecular refinement results.

## 1. Introduction

Refinement of macromolecular models is driven by both the experimental data and restraints. One universally applied form of restraints are geometry restraints, which provide *a priori* chemical information about the structural components of macromolecules. This chemical information about components is expressed with ideal (or target) values for bonds, valence angles, torsion angles, planes and chirality (Evans, 2007 ▸), along with estimated standard deviation (e.s.d.) values that represent the uncertainty of these targets. All refinement programs rely on restraints to produce chemically reasonable models. Restraints for common entities are stored in libraries that are used by most of the refinement programs. Commonly, the Engh and Huber library (Engh & Huber, 1991 ▸, 2006 ▸, 2012 ▸) is used for amino acids, and target values from Gilski *et al.* (2019 ▸) and Clowney *et al.* (1996 ▸) for nucleic acids. For example, *phenix.refine* (Afonine *et al.*, 2012 ▸), *BUSTER* (Bricogne *et al.*, 2017 ▸) and *REFMAC* (Murshudov *et al.*, 2011 ▸) (until 2018) use the Engh and Huber restraints or derivatives of them.

Macromolecular structures generally contain protein and/or RNA/DNA. Both of these are polymers described by the restraints libraries as individual sets of restraints for each of the amino acids and nucleic acids. The polymerization procedure in each program applies restraints to connect each unit in the chain. Another polymer class comprises carbo­hydrates, which also have units with restraints and a procedure to apply the appropriate links. Non-polymer entities include ions (mostly single metal ions coordinated to other parts of the macromolecule), metal clusters (also coordinating) and ligands (generally noncovalently bound molecules). Metals often adopt particular geometries with coordinating residues or form clusters with other metals and waters which benefit from restraints in refinement (Moriarty & Adams, 2019 ▸; Bhowmick *et al.*, 2023 ▸). However, it is algorithmically challenging to determine bonds and angles involving metals, and their coordination is further limited by a fundamental shortfall of the overarching restraint formalization (Moriarty & Adams, 2019 ▸). Therefore, metals and metal-containing entities are not the scope of this work.

The main topic of this work is ligands, but nonstandard amino acids are also considered. For the purposes of this study, ligands are molecules that contain at least two atoms and are not classified as polymers or ions.^1^ As of April 2025, more than 75% of models in the Protein Data Bank (PDB; Burley *et al.*, 2019 ▸; Berman *et al.*, 2003 ▸, 2007 ▸; wwPDB Consortium, 2019 ▸) contain at least one ligand. Restraints for ligands can be obtained in two ways: restraints for known ligands can be found in libraries (for example HEM and ATP), and those for novel ligands can be obtained from dictionary generators such as *AceDRG* (Long *et al.*, 2017 ▸), *Grade* (Smart *et al.*, 2011 ▸) or *eLBOW* (Moriarty *et al.*, 2009 ▸).

Although conceptually similar, libraries of ligand restraints face challenges compared with libraries of standard amino acids or nucleic acids. Firstly, there are many more ligands than standard (or common nonstandard) amino acids. For example, the PDB currently contains over 42 000 non-polymer entities (ligands) corresponding to over 90% of the Chemical Component Dictionary (CCD; Westbrook *et al.*, 2015 ▸) entries (the remaining 10% correspond to polymerizable entities or metal clusters; the ligand percentage will only increase as non-polymer additions will outpace polymer additions by a wide margin). Secondly, the composition of a ligand library needs to be constantly updated. That is, with each deposited model that contains a novel ligand, a new entry needs to be added to the ligand library. Adding restraints for new components is complicated by the fact that the PDB does not currently provide the restraints for the entities, only the bonding information and coordinates, and these do not necessarily represent ideal or representative geometries of the molecule. Thirdly, the development of new therapeutics is exploring new regions of chemical space. This leads to never before seen moieties that can cause issues with any restraint-generation technique. Lastly, many ligands occur only in a single instance of a PDB entry. Indeed, approximately 73% of the CCD entries only occur in a single deposited model, as opposed to standard amino acids (GLY, with nearly 230 000 PDB entries), ions (MG, ∼25 000), carbohydrates (NAG, ∼11 000), nucleic acids (U, ∼8000) and the most common ligand, HEM (∼6000 instances). Even for those restraints that occur in less than a few PDB entries, it is desirable that their generation be as robust and accurate as possible.

The Monomer Library (Vagin *et al.*, 2004 ▸; Murshudov *et al.*, 2011 ▸) is a restraints library that contains restraints for amino acids, nucleic acids, carbohydrates and common ligands. Versions of it have been used by several structural biology software packages, such as *REFMAC* (Murshudov *et al.*, 2011 ▸) in the *CCP*4 suite, the *Phenix* suite (Liebschner *et al.*, 2019 ▸) and *TNT* (Tronrud *et al.*, 1987 ▸) or *BUSTER* (Bricogne *et al.*, 2017 ▸) from Global Phasing. Each program uses the ideal and e.s.d. values to construct a complete internal representation of the restraints. For example, the program will look up the ideal bond length between two atoms of a model based on their residue and atom names. This means that every instance of this bond, such as the C^β^—C^γ^ bond in an arginine residue, will have the same ideal value. Polymerization of the amino acids, nucleic acids and carbohydrates is performed algorithmically using other information contained in the library.

*Phenix* originally adopted a trimmed version of the Monomer Library that retained a minimal set of restraints including those for amino acids, nucleic acids, small molecules and carbohydrates. Other most prevalent ligands were also included. The reason for retaining only a limited number of entries in the library was that the torsion restraints, either the ideal values, e.s.d. values or the periodicity, were often not sufficiently validated. Torsion restraints describe the conformations of a ligand, or puckers. With *Phenix* tools applying these restraints, it was necessary to adopt a library where these restraints are as correct as possible. The lack of validated torsion restraints applied particularly to carbohydrates, but also to nucleic acids and, to a lesser extent, amino acids (for example the H atom in NH_2_ groups).

Therefore, we introduced a new restraints library in *Phenix* designated the Geometry Standard (GeoStd) to house updated versions of the restraints. The GeoStd started with improved restraints for the standard amino acids that included the Rotamer Library (Lovell *et al.*, 2000 ▸) and updates of it (Hintze *et al.*, 2016 ▸). Ideal values for both amino acids and nucleic acids were adjusted, as were the restraint constructs such as nonperiodic torsion values. This involves the addition of a feature in the torsion restraints that allowed the listing of ideal torsion values rather than relying solely on the periodicity feature. This is particularly important in rotamers as not all of the periodic wells are actually minima. An example is the χ^2^ dihedral of tryptophan, which exists in the 0°, 90° and −90° configurations but not 180°. This scenario cannot be described with periodicity, so the capability to allow discrete values is an advantage. As rotamers are currently only available for standard amino acids, the alternative torsion feature is not automatic in restraints generation since all of the standard amino-acid restraints were hand curated.

Other examples of improvements of the GeoStd are the inclusion of arginine restraints that accounted for the asymmetry of the side chain and the deviation of the C^δ^ atom of the guanidinium moiety (Moriarty, Liebschner *et al.*, 2020 ▸), iron–sulfur cluster restraints and linkage to protein improvement (Moriarty & Adams, 2019 ▸), protonation-specific restraints for histidine (Moriarty, 2024 ▸) and using quantum mechanics (QM) to generate accurate geometries for radicals (Liu *et al.*, 2022 ▸). Recent improvements of the Engh and Huber values (Engh & Huber, 2012 ▸) are also implemented (Moriarty & Adams, 2021 ▸). The GeoStd initially contained just over 500 entries. This work expands the library to 38 140 entries.

Another example of updated restraints is the conformation-dependent library (CDL; Moriarty, Tronrud *et al.*, 2014 ▸; Moriarty, Adams *et al.*, 2014 ▸; Moriarty *et al.*, 2016 ▸), which allows backbone bond lengths and angles to vary with backbone conformation. The nuance with this approach is that the ideal and e.s.d. values are applied dynamically, dependent on the local geometry of each residue. Instead of applying a single target value from the standard restraints libraries, the values vary with the φ and ψ torsion angles of the protein backbone. *Phenix* has also recently added an implementation of the nucleic acid geometry conformation-dependent library RestraintLib (Kowiel *et al.*, 2016 ▸, 2020 ▸; Gilski *et al.*, 2019 ▸), another configuration-dependent library that includes functionals for more fine-grained values.

Complete descriptions of constituent molecules in experimentally determined 3D macromolecules in the Protein Data Bank (Westbrook *et al.*, 2015 ▸) are contained within the Chemical Component Dictionary (CCD). While each entry is stored in CIF format (Crystallographic Information Format; Brown & McMahon, 2002 ▸), it does not contain restraints. Among other information, the entries contain up to two sets of Cartesian coordinates (the first instance of deposited coordinates as well as an ideal calculated geometry), atom names, a bond list and several SMILES specifications (Weininger, 1988 ▸). Other fields include the entity name, ligand code, entity parent and date updated, to name a few.

**Figure 1:**
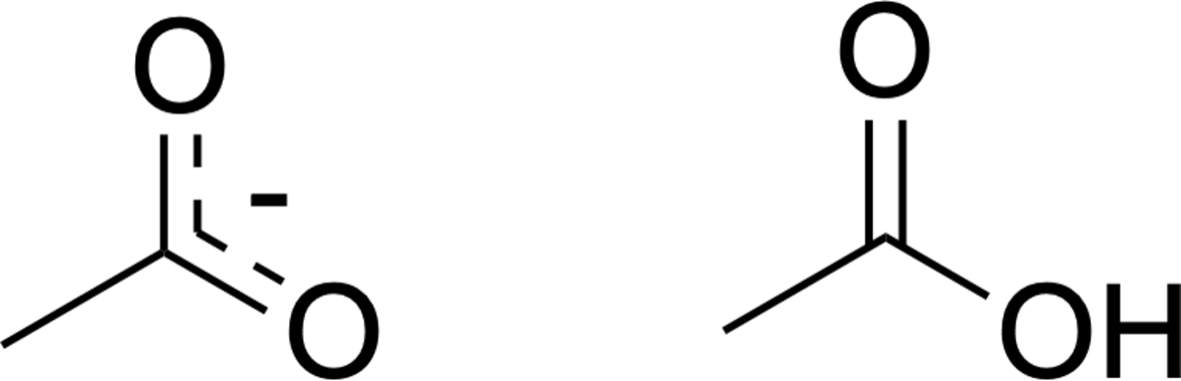
An example of an unprotonated (left) and protonated (right) acid: acetate and acetic acid.

Ideally, a general ligand restraints library would provide restraints for all entities in the CCD. This would make it straightforward to refine any model deposited in the PDB, without the need to generate restraints. However, as the content of the CCD is constantly updated, such a library would require constant updates as well. It is unrealistic that such updates can be maintained manually. On the other hand, it is challenging to automatically compute accurate restraints for new or updated ligands. The vast chemical space can result in moieties that have not been encountered before, including novel element combinations, protonation, charges and radicals. QM minimization relies on correct charge, protonation and radical state as a requirement. If these requirements are not met the minimization can converge poorly or fail while the resulting geometries can be poor or wrong. A simple example of inaccurate geometry arises at an acid moiety as in Fig. 1 ▸. If the acid moiety is not protonated (left side) in the experiment, but protonated in the QM calculation (right side), the resulting bond lengths and bond angles will be inaccurate. For these reasons, ligand restraints had been added *ad hoc* to the GeoStd before this study was undertaken.. Many of the *ad hoc* entries were calculated with QM methods and manually validated in various ways.

As the generation of ligand restraints is a common bottleneck for users of crystallographic software, we decided to significantly increase the number of ligand entries in the GeoStd and to standardize and streamline the addition of novel restraints. This work describes the procedure used to add more ligand entries to the GeoStd restraints library. We describe how entries to be added were selected, how the restraints were generated and how the minimized geometries were validated.

## 2. Methods

To add new entries in the GeoStd, we first determined suitable targets from the CCD (Section 2.1). We then calculated geometries using quantum-mechanical methods (Section 2.2) and generated restraints (Section 2.3). The minimized geometries were then validated (Section 2.4). Finally, we also added the functionality of creating force-field files for the *Amber* molecular-mechanics software (Section 2.5).

### 2.1. Calculations

All polymer and non-polymer entries in PDB entries are contained in the CCD. As described above, the information stored in the files in this database does not correspond to that present in restraint files, although the file format (CIF) is the same. We used the following information stored in these CIF files: the canonical SMILES string of the molecule, the list of atoms and the list of bonds. Specifically, the list of atoms defines their names, which are used to match ligand atoms in a model with the corresponding restraints in a library. Each CCD entry has a second list of legacy atom names if they have been updated since the creation of the initial entry. This may occur during periodic remediation operations performed by the PDB, such as one effort focused on carbohydrates (wwPDB; https://www.rcsb.org/news/5f17634f0b582626e650e48b) and another effort in 2007 that changed the H-atom names of amino acids (known as v2 to v3 name conversion).

The version of CCD on 30 July 2024 contained 44 095 entities, including all amino acids, nucleotides, saccharides, ions, metal clusters and non-polymers (ligands).

As a first step, the entities in the CCD were filtered. We excluded standard amino acids and nucleic acids, as their restraints are already established and validated. Furthermore, we ignored single-atom entries (mostly metal ions, or inert gases, *e.g.* PDB entry 7q38) because metal coordination is not within the scope of the GeoStd. We also filtered ligands incorporating metals or metal clusters (*e.g.* HEM or SF4). To perform QM calculations, metal entries require details of the metal charge and multiplicity (electron distribution in the orbitals). The multiplicity is not provided by the CCD and the charge often changes upon interaction with receptors, so the restraints would not be generally applicable. Furthermore, higher levels of QM are required for accurate computations involving metal bonding.

In addition, we ignored entries that were already in the GeoStd and that had passed previously applied validation. We also did not reprocess manually edited entries, such as the radical MTN, for which the plane restraint was adjusted using *REEL* (Moriarty *et al.*, 2017 ▸) to account for the added flexibility of the radical moiety (Liu *et al.*, 2022 ▸), or CLA, for which different planarity restraints for low-, medium- and high-resolution regimes were created and contributed by Dale Tronrud. Any CCD entries denoted as obsolete were also ignored.

To update restraints efficiently, we also checked whether a CCD entry had changed significantly since its initial addition to the GeoStd. As an example, when the atom names or the SMILES string of an entry in the CCD had been updated, they were accordingly updated in the GeoStd entry. If the molecule content in the CCD was changed compared with what was present in the GeoStd, the corresponding entry was recreated using the procedure described below.

Our work on a new implementation of joint X-ray/neutron refinement (Liebschner *et al.*, 2019 ▸) highlighted the need to support different versions of restraints for a ligand, *i.e.* to support all possible protonation states of PDB entities. By default, *eLBOW* generates restraints for typical pH levels in the protein environment. This means, for example, unprotonated acid moieties. However, protonated acid groups are often detected in neutron refinement. The pipeline used to generate the restraints in this work determines whether a moiety may be protonated and computes all minimized geometries and corresponding restraints. Accordingly, the restraints lookup in *Phenix* has been changed to load the appropriate restraints based on the model contents.

Notwithstanding that models can contain acid moieties that are either protonated or not protonated, they are usually deprotonated in proteins *in situ*. By contrast, the information for each CCD entry is generally the protonated state. As a consequence, the database – *notitia collectis*^2^ – can have records that are different (in protonation state) to the *in situ* state. We designate the molecule in the CCD as *in notitia* and the molecule most likely to be in the protein model as *in situ.*

After filtering, the majority of remaining components are non-polymer molecules, for which it is generally easier to calculate minimized geometries as they are not covalently bound to other entities. For these components, the environment can be approximated by the use of solvent models and the generation of *Amber* force fields is simplified.

Some additional steps are needed for polymerizable components. For example, nonstandard amino acids in the CCD have neutral moieties on the C- and N-termini, which is not the situation in the midst of protein (peptide plane) or at the termini of the protein chain (charged). The neutral entity is helpful for QM calculation convergence and a semblance of chemical realism, but is not a representative geometry of the sample. The restraints are adjusted as discussed in Section 2.3.

### 2.2. QM methods

After filtering the CCD entries to obtain ligands to add to or update within the GeoStd, the geometry of each molecule in the subset was minimized using quantum-mechanical methods.

Thus far, restraint generation has generally been based on experimental data. Quantum mechanics is an orthogonal alternative to using experimental structures, allowing the calculation of geometric parameters from first principles, rather than relying on statistical averages from a diverse population of structures. While CSD-derived ideal values are indeed powerful due to their statistical validation against a vast repository of high-quality experimental data, they are population averages. These values represent the most probable geometry for a chemical fragment as observed within the constraints of a crystal lattice. However, the CSD approach is limited by the availability and diversity of the collected data. For novel or rare chemical scaffolds, there may be insufficient data to derive statistically meaningful restraints. Furthermore, the distributions of the values in the CSD can deviate greatly from Gaussian and can, on occasion, have bimodal distributions. QM provides a robust method for generating accurate restraints for any molecule, regardless of whether it has been previously synthesized or crystallized.

A plethora of QM methods are available to predict the behaviour of molecules, with the methods having varying levels of sophistication. The challenge is that the choice of the method and level of sophistication needs to balance the required accuracy of the result (such as the minimized geometry of a molecule) and the computational cost. There are two major sticking points in QM that require increasing computer resources to improve accuracy:

(i) Electron-correlation issues arise because the motion of electrons is not independent; they repel each other (Coulomb correlation) and must obey quantum symmetry rules (Fermi correlation), making exact solutions impossible for all but the simplest systems, leading to complex ‘many-body’ calculations requiring advanced approximations to capture essential chemical behaviour.
(ii) Basis sets are mathematical constructs used to represent the spatial electron distribution in the molecule. Issues with this formalism involve balancing computational cost resulting from increased numbers of mathematical functions with accuracy.

The entity geometries were minimized using two QM methods taken from a comprehensive evaluation (Goerigk *et al.*, 2017 ▸) of the ‘zoo’ of techniques available. The first, PBEh-3c (Grimme *et al.*, 2015 ▸), generates accurate geometries, while the second, more computationally expensive, B3LYP D3BJ TightSCF RIJCOSX def2/J def2-TZVP, known as ωB97X-D3 (Lin *et al.*, 2013 ▸), was determined to be one of the best methods available. The first method prescribes a basis set, but the second (ωB97X-D3) can be paired with various basis sets, with the chosen triple-ζ basis set being a moderate-level choice. A possible third-rung QM method could be the ωB97X-D3 method paired with a larger basis set such as QZTP that uses four ζ functions.

Both QM minimizations performed employ the CPCM solvent treatment (Barone & Cossi, 1998 ▸) to simulate more closely the environment in the protein (crystal) with the added benefit of preventing electrostatic moieties from generating spurious geometries. If the PBEh-3c method failed validation, the second method was used. The PBEh-3c method is considered a ‘high-speed’ method, but at 17 h on a modern MacBook Pro (Apple Silicon M1Pro with 32 Gb memory on a single core) the time to minimize ATP is greater than the average user is willing to wait. Normalizing this timing to a per-optimization step value gives 61 min. The per-step value for ωB97X-D3 is 242 min. It should be noted that the timings are influenced by the number of atoms (*N*) scaling as O(*N*^3^), meaning that doubling the number of atoms (42 for ATP) means eight times the wait. Both sets of calculations were performed using the *ORCA* software package (Neese *et al.*, 2020 ▸). A semiempirical QM method, PM6-D3H4 (Řezáč *et al.*, 2009 ▸), that has a per-step timing of 0.27 s was also assessed for suitability due to its computational efficiency and ease of use via *MOPAC* (Moussa & Stewart, 2024 ▸) as distributed with *Phenix*. These four QM levels can be considered a suitable series of increasing accuracy with a resultant increased computational cost.

### 2.3. Restraint generation

Once the minimized molecular geometries of the ligands were obtained with QM methods, the *eLBOW* program was used to generate restraints using the ideal values from the minimized geometries and the e.s.d. values from the validation step (see Section 2.4). This allowed adjustment of the dihedral restraints type and redundancy. In particular, the restraint-generation mechanism in *eLBOW* ensures that all H-atom restraints are complete. This is relevant for refinement, because *phenix.refine* uses a riding hydrogen model (Liebschner *et al.*, 2020 ▸) to maintain the ideal positions of H atoms during refinement. The method relies on bond lengths, bond angles and torsion restraints to position the H atom.

Some entities, such as standard amino acids, primarily exist in polymerized form. Polymerization changes the chemical details of the entities, so the restraints need to be modified compared with the single-entity versions. While standard amino acids were not reprocessed in this work, polymerization can also occur for other entities, such as nonstandard amino acids. QM minimization was performed on the neutral entity as it is present in the CCD but, to obtain appropriate targets that facilitate automatic polymerization in *Phenix*, the superfluous atoms at the termini involved in polymerization (H2, OXT and HXT) were removed from the restraints. We note that if such a polymerizable moiety occurs at a terminus, correct termination (such as adding back the H2 atom) can be handled algorithmically in *Phenix*.

Recently, the PDB moved to allow the description of ligand IDs with five characters (instead of three) to account for the growing number of polymer entities (https://www.rcsb.org/news/63ff72ccc031758bf1c30ff7). With *Phenix* tools being able to process these entries, our approach was also applied to ligands with five-character IDs. Accordingly, the resulting new version of GeoStd includes 505 components with a five-character ID.

### 2.4. Validation

The program *Mogul* (Bruno *et al.*, 2002 ▸; Macrae *et al.*, 2008 ▸) in the Cambridge Structure Database (CSD) suite (Groom *et al.*, 2016 ▸) allows validation of the ligand geometries. It uses accurate experimentally determined small-molecule geometries from CSD entries to provide ideal and standard deviation (s.d.) values for bonds and angles which can be used to validate the QM geometries before being added to the GeoStd. Torsions and rings are validated by *Mogul*, but the focus in model refinement is the bond and angle r.m.s.d. values. The torsion ideal and periodic values are determined using a simple empirical approach involving hybridization of the constituent atoms. Validation of rings and the determination of torsion parameters is outside the scope of this work.

To validate the ligand geometries based on the *Mogul* s.d. values, we used *Z*-scores. The *Z*-score measures by how many standard deviations a specific metric (a bond length, a bond angle *etc.*) deviates from its ideal value. For example, an angle *Z*-score of 1.0 means that a particular angle deviates by one standard deviation from the ideal value given by the above *Mogul* analysis. We note that the PDB validates ligands using the *Z*-scores of the bonds and angles, using the *Mogul* s.d. values to calculate the root-mean-squared *Z*-score (r.m.s.*Z*) of the whole ligand, and lists all the bond and angle r.m.s.*Z* values greater than 2, in keeping with the recommendations of the ligand taskforce (Adams *et al.*, 2016 ▸) that the r.m.s.*Z* be less than 2. PDB validation reports highlight ligands with r.m.s.*Z* values greater than 2 and also include a percentage of bonds and angles with individual r.m.s.*Z* values great than 2. Experience has shown that the s.d. values from *Mogul* are generally too small for practical purposes as e.s.d. values in refinement at resolutions typically achieved in macromolecular crystallography. It is more appropriate to use twice the *Mogul* s.d. values for e.s.d. values in refinement (Moriarty & Adams, 2019 ▸; Moriarty, Liebschner *et al.*, 2020 ▸; Moriarty, Tronrud *et al.*, 2014 ▸).

We adopted the following validation scheme using the maximum *Z*-score. If the maximum (absolute) *Z*-score was less than 2, 4 or 6, the geometry was designated as ‘awesome’, ‘superior’ and ‘satisfactory’, respectively.

As noted above, *Mogul* generally provides small s.d. values if there are only a few moieties of one type. This is particular true with the strict options used in this work (see the supporting information for an example). Further, an extreme scenario occurs when there is only one example of a specific bond or angle in the CSD. This can result in an s.d. value of zero and validation failure. To include such geometries, *ad hoc* minimum s.d. values for bonds and angles of 0.005 Å and 0.75°, respectively, are applied. These values are quite conservative as they are approximately half the normal s.d. values. If the validation passes using these *ad hoc* s.d. values the geometries are designated ‘satisfactory (reasonable std)’ (no matter the passing *Z*-score).

Another special case exists for polymerizable entities. The polymerization of the amino acids means that the ideal values of bonds and angles in the polymer backbone are different from the isolated entity. It follows that the bond and angle ideal values can be updated in the restraints based on the parent type of the amino acid without the need for validation. However, validation is required for the side chain. A geometry validation that is side-chain specific has ‘(side chain)’ added to the designation. It should be noted that any *in notitia* geometries are only validated once the *in situ* geometry passes validation.

### 2.5. Force-field files

Macromolecular refinement results can be improved using molecular-mechanics force fields such as those in *Amber* (Case *et al.*, 2023 ▸; Moriarty, Janowski *et al.*, 2020 ▸). Accurate non-polymer geometries can be used to generate *Amber* force fields to streamline *Amber*-based refinement. While a tool exists in *Phenix* to generate force-field parameters based on a model, the presence of curated force fields aids in the speed and accuracy of *Amber* refinement. In addition, the *Amber* force fields can also be used in other calculations such as molecular mechanics and molecular dynamics.

For each ligand structure, we used the *antechamber* and *parmchk*2 programs in *AmberTools* (Case *et al.*, 2023 ▸) to prepare force-field files using the general *Amber* force field (GAFF2; Wang *et al.*, 2004 ▸) and the ABCG2 atomic charge model (Sun *et al.*, 2023 ▸; He *et al.*, 2025 ▸). Since the input geometries had already been minimized at a QM level, the charge calculation used the QM geometries directly, omitting a minimization using a force-field method. We also filtered out entities that GAFF2 cannot handle: elements other than H, C, N, O, F, Cl and I, components that are parts of polymers, and the *in notitia* entries with protonated carboxylic acids. This led to force-field files for 37 788 components. Each component was then minimized in the resulting force field to assess how far the force-field optimized geometry deviates from the input, QM-optimized geometry. This was performed using the gb = 1 generalized Born solvent model and a monovalent salt concentration of 0.1 *M*. It should be noted that these minimizations affect only subsequent *Amber* uses and the actual value has little influence on the results.

Although the *AmberTools* programs were used to generate the force-field files, the results can be used in many other molecular-simulation programs. The *ParmEd* facility in *AmberTools* facilitates transfer to other common simulation programs such as *OpenMM* (Eastman *et al.*, 2024 ▸), *GROMACS* (Páll *et al.*, 2020 ▸) and *CHARMM* (Brooks *et al.*, 2009 ▸).

## 3. Results

Of the 44 095 entries in the CCD, 42 150 remained after filtering as starting points for QM minimizations. QM minimization at the PBEh-3c level resulted in 40 342 optimized geometries. Of these, 32 849 passed validation (81%), as seen in the ‘PBEh-3c’ column of Table 1 ▸, the majority with the ‘superior’ designation. The ‘awesome’ and ‘satisfactory’ categories also have a large number of entries. We note that a significant number of components were assigned to the ‘reasonable std’ category, indicating that many *Mogul* s.d. values were too small. We also note that the ‘side chain’ classification is a small fraction of the total.

The second QM level was applied to the 16% (6381) of filtered entries that had not passed the first round of validation. This time, 4833 (76%) entries passed the validation, with the majority of entries having either ‘awesome’ or ‘satisfactory’ geometry. We note that once again the application of *ad hoc* s.d. values added a significant number of entries. The set of calculations is restricted to the failed entries from the previous QM level, which may explain the reduced success rate (76% versus 84%). In total, 37 682 restraints files were added to the GeoStd using the validated QM geometries.

As noted, restraints were also calculated for *in notitia* protonation states of the ligands and included in Table 1 ▸. This procedure checked whether a protonated state exists for a ligand before expending resources. It was also only performed using the PBEh-3c method. The table shows that 6082 ligands have protonated restraints that passed validation, with the majority having an ‘awesome’ classification.

**Figure 2:**
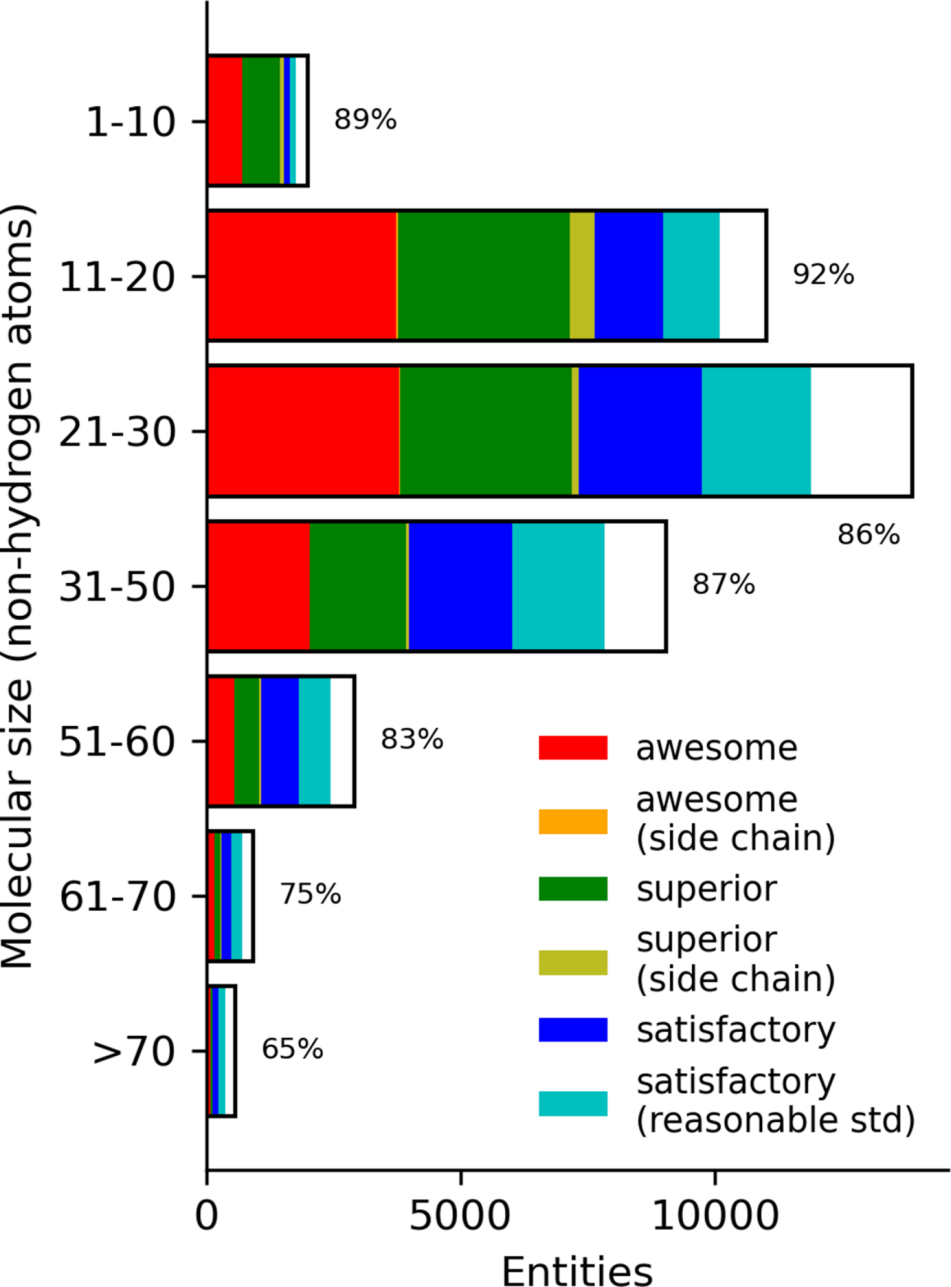
Number of PBEh-3c geometries calculated for each molecular size binned with a width of 10. Colours in the bar indicate the number of validation classifications per the legend, with the white portion representing the fail classification. Note that the orange bar is barely visible but evident. The total success rate is displayed next to each bar.

The size of the molecules has a nonlinear impact on the efficiency of the calculations. Even the simplest QM methods scale as the cube of the number of atoms, with more complex QM scaling at higher powers. Many techniques are employed to lower the scaling, but they generally use a reasonable interaction cutoff for larger molecules. The sizes of entities calculated by the PBEh-3c QM method are displayed in Fig. 2 ▸, with the raw data in Supplementary Table S1. The information is binned in groups of ten non-H atoms. The majority of molecules are smaller than 60 atoms, which falls within the 20–70 range as delineated by Ghose *et al.* (1999 ▸) for the typical molecule size of a drug candidate.

The proportion of the validation categories are shown as different colours in the bar, with the white portion indicating those geometries that failed *Mogul* validation. The majority of geometries in the drug-like size regime pass *Mogul* validation, with the smaller molecules being more likely to be accurate. Smaller molecules (1–10) had 29% in the ‘awesome’ classification, while large components (>70 atoms) had only 12%. We observe a similar behaviour for the ‘superior’ classification. Unsurprisingly, the ‘satisfactory’ classifications increase as a fraction of the total as the molecular weight increases (6% for 1–10, increasing to 20% for 41–50). The ‘reasonable std’ group is about the same as the unmodified e.s.d. group in all molecular-size bins.

**Figure 3:**
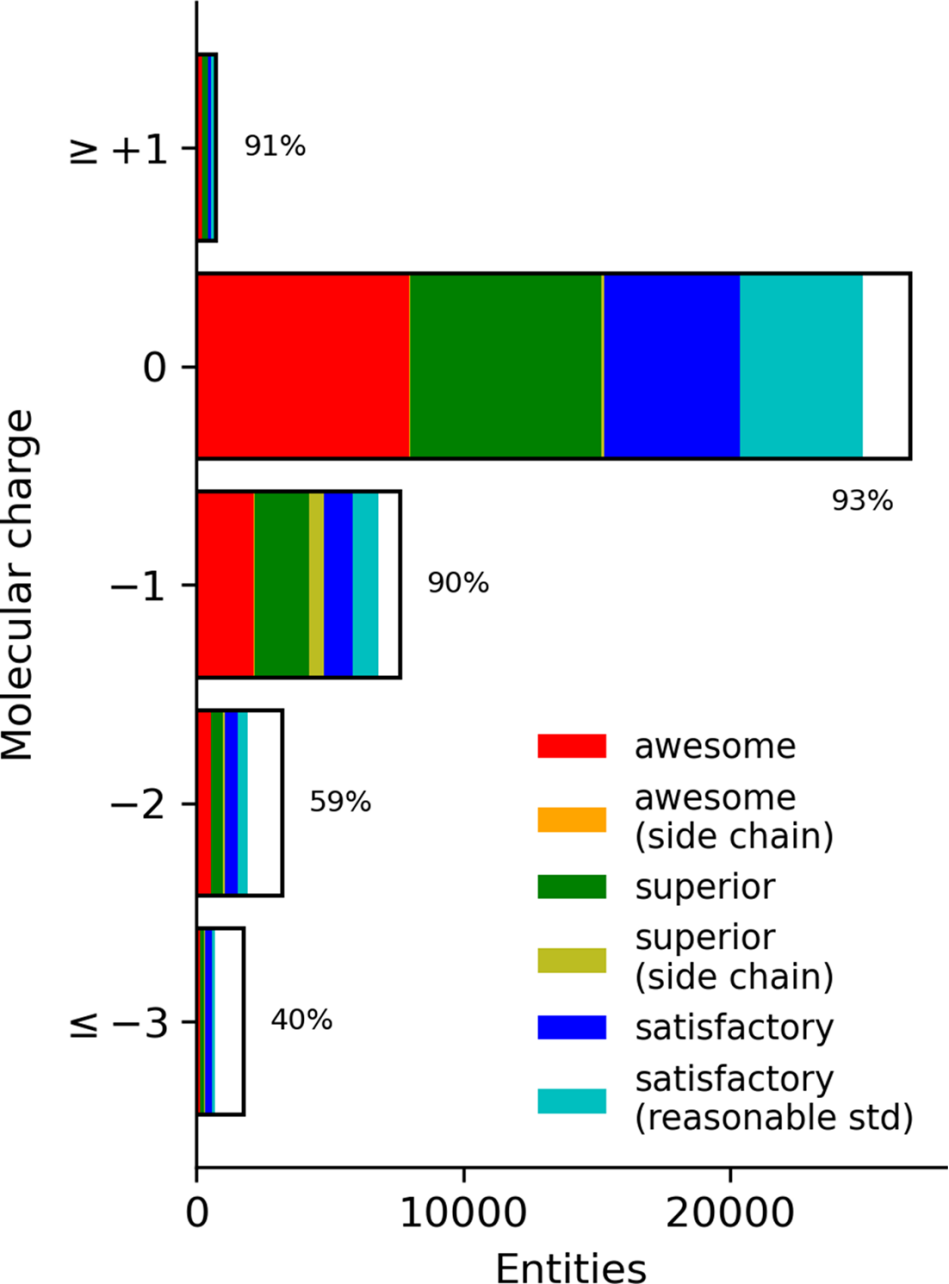
Number of calculated molecules with charge. Colours in the bar indicate the number of validation classifications per the legend, with the white portion representing the fail classification. Both of the (side chain) bars are barely visible but evident.

Specific moieties are another characteristic of a molecule that influences the outcome of a QM calculation. This can be investigated by the molecular charge and element composition. The distribution of the molecular charge of the PBEh-3c geometries is shown in Fig. 3 ▸ (raw data are given in Supplementary Table S2). Unsurprisingly, the majority of the entities are neutral, as is the policy of the CCD. However, because the process used to generate restraints automatically deprotonates acid moieties, there is also a significant number of negatively charged ligands.

The validation success rate of the singly charged molecules is nearly 90%, while neutral and positively charged molecules are in the 70% range. More negatively charged molecules have lower success rates.

**Figure 4:**
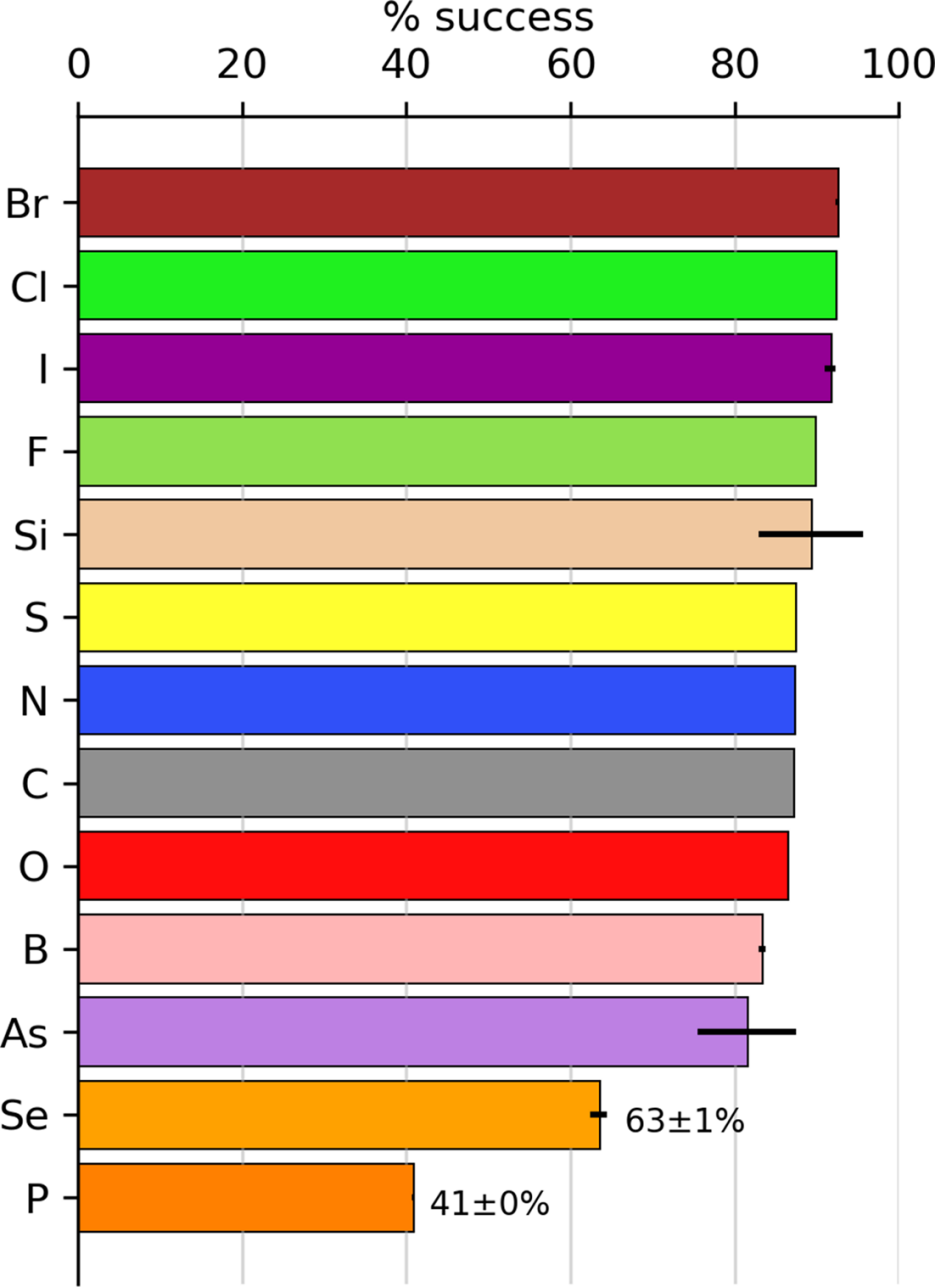
Number of calculated molecules with charge. Colours in the bar indicate the number of validation classifications per the legend, with the white portion representing the fail classification. The success rate of molecules containing a given element is shown using the CPK convention. Error bars are determined by the population of entities, with the given element resulting in vanishingly small values. The values of success and error estimates are included for the lowest two elements.

The element content of a molecule can affect the validation outcome for a calculated geometry. It is also a proxy for moieties that can be difficult to minimize to a geometry that will pass validation. Fig. 4 ▸ shows the validation success rate based on the element content of the molecule. Molecules with halogens had the highest success rate (Br, 92%; Cl, 92%; I, 92%; F, 90%). Note that error bars are included based on population, with some bars being too small to be displayed. Molecules containing C, N and O atoms (the bulk of the molecules) have a rate of approximately 87%. The lowest significant success rate was for molecules containing P atoms at 41%. These molecules are of particular note because the phosphorus is often in a phosphate moiety, with the charge on the O atoms causing problems for the QM calculations. Alternatively, it may be because many phosphates in the PDB and CSD are coordinated to a magnesium ion. The presence of the ion greatly affects the bond lengths and angles of the coordinating molecule that are not reflected in the QM calculations of the isolated entity.

An example of a phosphorus molecule containing a bond outlier is CCD ID 1sH.^3^ The bond length for the P1—O2 bond is predicted to be 1.623 ± 0.009 Å by *Mogul* using the options in the supporting information. It should be noted that including organometals changes this value to 1.608 ± 0.043 Å: both a different ideal value and a greatly increased variation. PBEh-3c minimized to 1.702 Å, while ωB97X-D3/TZVP gave 1.681 Å, which is just greater than a *Z*-score of 6 (but within the range for metal interactions). A geometry calculated using a QZVP basis set (a possible third-rung QM method) predicted a bond length of 1.674 Å, within the ‘satisfactory’ classification. This demonstrates that the QM hierarchy improves the agreement with experimentally determined values. In fact, there is no significant difference in using either set of restraints when refining the 1.72 Å resolution structure PDB entry 4KRi that is the sole model that contains 1sH.

The presence of selenium in a molecule also appears to present some problems. We can hypothesize that selenium, as a heavier element, has known challenges for standard quantum-mechanics methods that are typically parameterized for lighter elements. However, there are only about 120 selenium-containing ligands in the CCD, so the impact is more limited.

The development of protonation-specific restraints resulted in nearly 6000 entries, as shown in the last column of Table 1 ▸. Table 2 ▸ shows the number of validation classifications of the *in situ* and *in notitia* states. The last column is the ligands that failed validation for the *in notitia* molecule. When the corresponding *in situ* ligand was calculated, 149 were classified as ‘awesome’, 15 as ‘superior’ and 405 as ‘satisfactory’. The likely reason that most of the *in notitia* ligands only passed as ‘satisfactory’ is because the *in situ* ligands were just inside the *Z*-score of 6 cutoff. The largest number, 1835, of ligands were classified as ‘awesome’ for the *in situ* protonation state and were downgraded for the *in notitia* calculations to ‘superior’. Clearly, the geometries are sensitive to the protonation state, but there does not appear to be a clear trend. The number of entries that passed validation only for *in situ* geometries was 569 (last column sum).

**Figure 5:**
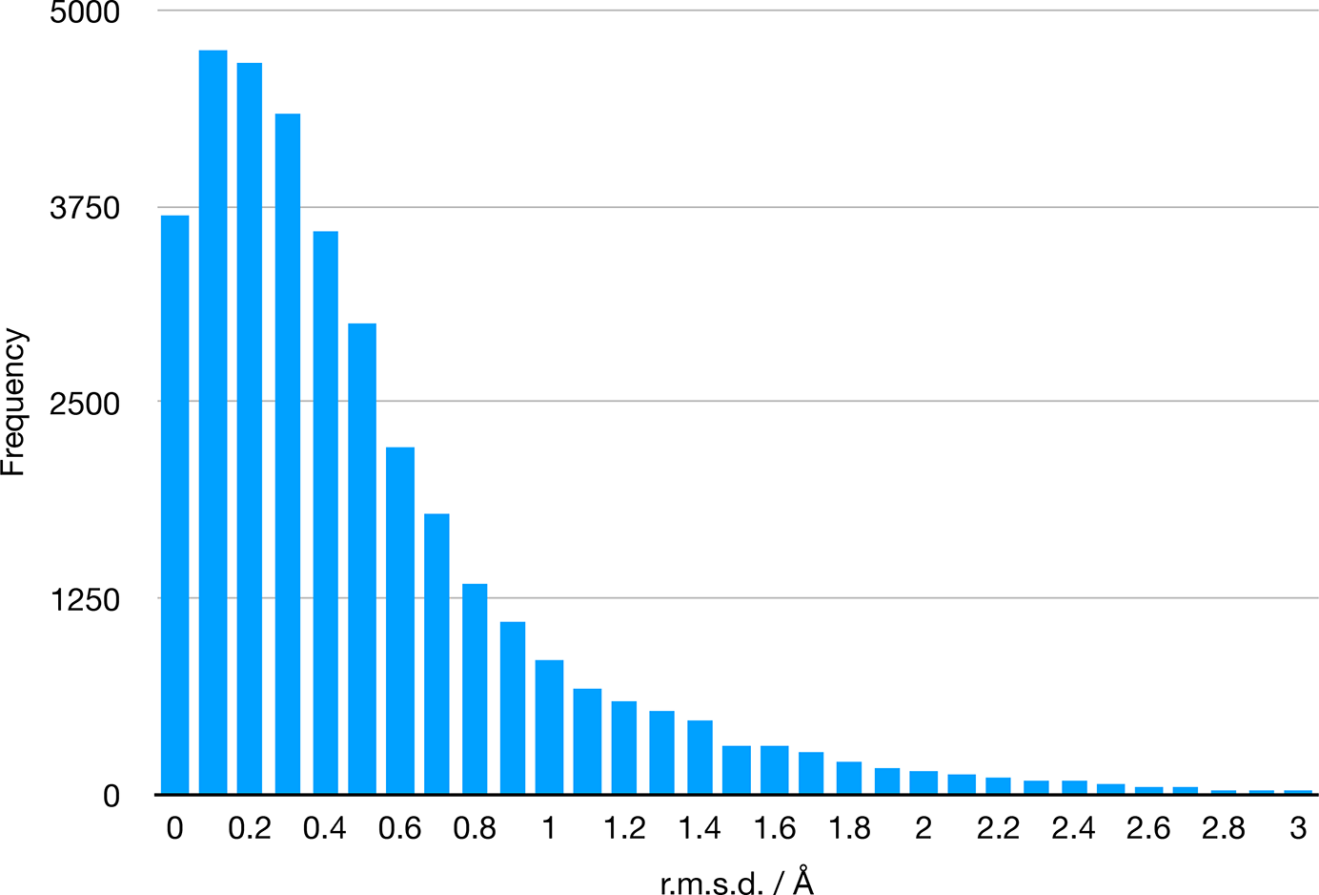
Differences between the starting and finishing geometry in r.m.s.d. of the *Amber* force-field parameter files.

As mentioned above, each of the components that were subject to force-field generation were then fully minimized to the nearest local minimum in *AmberTools*. Fig. 5 ▸ shows how much (as measured by r.m.s.d.) the MM-optimized structures deviated from the starting QM-optimized structures. Nearly 60% of the components changed less than 0.5 Å upon MM minimization, while 85% changed less than 1 Å. Many of the molecules that changed more than this contain one or more rotatable bonds, with the MM optimum being in a different rotameric state. These cases do not lead us to believe the QM-minimized geometry is necessarily inaccurate. The minimized structures and optimization details are also in the library, allowing users to assess the behaviour of any particular component of interest.

**Figure 6:**
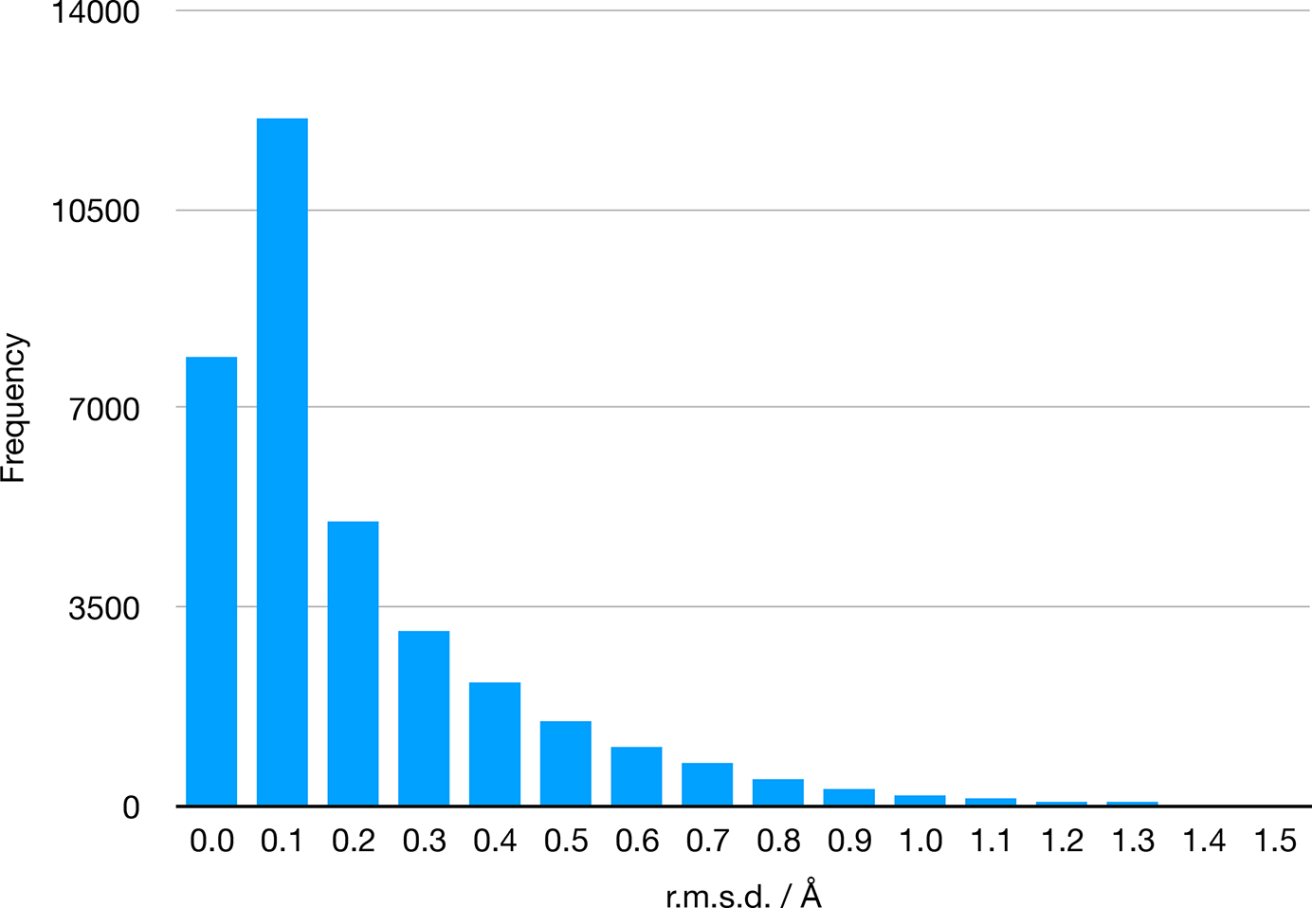
Differences between the starting and finishing geometry in r.m.s.d. of the restraints files.

A similar validation was performed using the geometry-minimization program in *Phenix* (*phenix.geometry_minimization*). For each ligand, a geometry minimization was performed starting with the ideal geometry from the restraints file. This procedure tests whether the restraints generated from the ideal geometry maintain that ideal geometry. The r.m.s.d. values between the starting and final geometry are shown in Fig. 6 ▸. Because the geometry-minimization algorithm has a far simpler nonbonded interaction, only a repulsive potential, the r.m.s.d. distribution is skewed more towards zero than the MM force-field minimizations. In fact, 87% are less than 0.5 Å and 98.2% are less than 1.0 Å. Rotatable bonds contributed to the larger r.m.s.d. values, for example CCD ID Fi3, but larger molecules tend to have larger r.m.s.d. values even when there are small deviations from the ideal restraints values that leverage the global measurement; for example, CCD ID 3ZH.

The semiempirical QM method PM6-D3H4 was also assessed for suitability due to its ease of use (distributed with *Phenix*) and computer-efficiency (Supplementary Table S3). The general observation is that the PM6 geometries have just over 26 000 validated geometries compared with PBEh-3c, with nearly 33 000. Furthermore, the majority of the successes are in the ‘satisfactory’ category compared with ‘awesome’. We conclude that while this level of QM has a lower success rate, it is highly computer-efficient.

## 4. Discussion

To improve on future iterations of this effort, we investigated entities not added to the GeoStd. Firstly, the analysis showed that a significant number of models in the PDB have unknown entities designated UNX (817) and UNL (550). Such molecules cannot be processed. Many of the next most populous entities are nucleotides, including DPo (diphosphate), and metal clusters, including CUA (dinuclear copper ion). Metal clusters can be determined via other methods, but an investigation into phosphate moieties is warranted as one third of the entities that failed validation contained phosphorus. The development of an effective procedure for nucleic acids and nucleotides would greatly reduce the outstanding restraints as well as increase understanding of the chemistry of the phosphate moiety. The ‘missing’ ligand restraints translates to less than 8% of PDB entries needing restraints beyond the GeoStd. This drops to 2.3% when ignoring RNA/DNA and metal entities, both of which are outside the scope of this work. It is likely that these are ligands of interest and, as such, deserve increased attention. It also highlights the need for the deposition of restraints into the PDB for subsequent download.

We note that QM geometry minimizations work well for molecules with solely covalent bonds, covering the majority of drug-like candidates. Metals were excluded from this study because QM methods need additional attention, including the specification of electron configurations and higher levels of method and/or basis sets. Although this study was focused on covalent molecules, we performed some tests with metals, which showed that even for metal-containing ligands without electron-configuration issues, magnesium and calcium, the validation success was 38% and 20%, respectively.

The integration of *MOPAC* (Moussa & Stewart, 2024 ▸) into the *Phenix* distribution has streamlined the use of semi-empirical QM for restraint generation. It is a complementary method to the use of *Mogul* for restraint generation. Furthermore, an interface with the *ORCA* software package allows the use of the same methods verified in this work. Restraints for novel entities with drug-like characteristics can be generated with confidence.

After generating restraints, it is important to validate the ligand geometry as protein refinement progresses. We note that one can submit the model to the PDB for a validation report directly from the *Phenix* GUI. This is particularly important if the ligand is important to the results and reduces the possibility of surprises when the model and data are deposited.

## 5. Conclusions

A procedure for generating and validating geometric restraints for ligand entities in the PDB Chemical Component Dictionary/Library (CCD) was developed and tested on over 42 000 entities. Each successfully validated set of restraints was added to the GeoStd, a freely available library of restraints in two formats (CIF and *Amber* force-field files) that can be downloaded from https://github.com/phenix-project/geostd. We note that this is also distributed with the *Phenix* software. Files relating to a particular CCD entry can be found at https://phenix-online.org/phenix_data/geostd/ by navigating to the appropriate directory.

The resulting restraint and molecular-mechanics files can be used directly for crystallographic refinement or molecular-dynamics simulations. The validation effort also illustrates the strengths and limitations of our current automated procedures for handling large numbers of chemical entities, such as small molecules and modified residues. Importantly, the restraints do not rely on the experimental geometry in the CSD directly. Using a validation method (in this case *Mogul*) to guide the refinement has challenges and can lead to a feedback loop that reinforces the possibly flawed metric. An independent method for generating restraints is a positive step to independently verify the obtained geometries. By design, the restraint-generation and validation procedures discussed in this work can be repeated periodically as entries are added to the CCD. Furthermore, higher levels of QM can be investigated to address issues with difficult moieties.

## Supplementary Material

Supplementary Tables and Schema. DOI: 10.1107/S2059798326000975/von5004sup1.pdf

## Figures and Tables

**Table 1:** Number of validation results in each category of the PBEh-3c (both *in situ* and *in notitia*) and ωB97X-D3/TZVP-calculated molecules

Validation class	PBEh-3c	ωB97X-D3/TZVP	PBEh-3c (*in notitia*)
Awesome	5566	1723	2801
Awesome (side chain)	64		16
Superior	15315	552	1171
Superior (side chain)	791	3	97
Satisfactory	6363	1186	1546
Satisfactory (reasonable std)	4750	1369	451
Total	32849	4833	6082

**Table 2:** Number of entry classifications for the unprotonated geometries that validated into each classification for the protonated geometries

	*In nototia*			
*In situ*	Awesome	Superior	Satisfactory	Fail
Awesome	313	753	77	149
Superior	1835	445	496	15
Satisfactory	519	55	1017	405

## Data Availability

The Geometry Standard (GeoStd, pronounced Geo Standard) restraints library is available in the *Phenix* suite distribution (versions 1.21.2 and later) and at https://github.com/phenix-project/geostd. Individual entries may be accessed at https://phenix-online.org/phenix_data/geostd/.

## Footnotes

1 The CCD has dinuclear metal pairs as ions. Furthermore, chloride is a nonmetal ion. These assignments are based on _chem_comp.type (https://mmcif.wwpdb.org/dictionaries/mmcif_ma.dic/Items/_chem_comp.type.html) and _chem_comp.pdbx_type (https://mmcif.wwpdb.org/dictionaries/mmcif_ma.dic/Items/_chem_comp.pdbx_type.html).

2 Latin. Other translations are possible – *theca datorum* or *tabula datorum* – but *notitia* translate to knowledge, notice or record, which fits well with the idea of a record for each entity.

3 PDB and CCD ID codes are written using the ‘human readable’ format as described in *Human Readable PDB Codes* (Moriarty, 2015 ▸).
