# Supplementary material for: Validated ligand geometries for macromolecular refinement restraints and molecular-mechanics force fields
Source: Acta Crystallogr D Struct Biol. 2026 Feb 18;82(Pt 3):216–26. doi: 10.1107/S2059798326000975 (PMC12954859; doi:10.1107/S2059798326000975)
Supplement: Supplementary file 1 [file d-82-00216-sup1.pdf]

Acta Cryst D STRUCTURAL BIOLOGY

Volume 82 (2026)

Supporting information for article:

Validated ligand geometries for macromolecular refinement restraints and molecular-mechanics force fields

Nigel W. Moriarty, David A. Case, Dorothee Liebschner and Paul D. Adams

Table S1 More details of the validation results as summarised in Table 1.

Size	*awesome*	*awesome (side chain)*	*superior*	*superior (side chain)*	*satisfactory*	*satisfactory (reasonable std)*	*fail*	*Total success*	*Total Calculated*
10	699	12	738	67	136	115	630	1767	2397
20	3725	36	3387	478	1353	1113	3899	10092	13991
30	3797	8	3388	134	2422	2129	5626	11878	17504
40	2033	6	1887	57	2032	1808	3273	7823	11096
50	556	0	481	42	733	620	1190	2432	3622
60	158	2	120	13	202	201	532	696	1228
70	58	1	28	1	55	98	258	241	499
80	13	0	7	1	28	23	78	72	150
90	5	0	8	1	14	12	31	40	71
100	1	0	3	0	6	7	20	17	37
110	0	0	1	0	2	1	9	4	13
120	0	0	0	0	1	3	6	4	10

Table S2 More details of the validation results as summarised in Table 2.

Charge	*awesome*	*awesome (side chain)*	*superior*	*superior (side chain)*	*satisfactory*	*satisfactory (reasonable std)*	*fail*
-3	7.40%	0.15%	6.75%	2.60%	11.64%	6.25%	65.22%
-2	16.85%	0.15%	13.92%	2.59%	14.38%	11.05%	41.07%
-1	28.08%	0.42%	26.56%	6.96%	14.40%	12.29%	11.30%
0	21.79%	0.06%	19.67%	0.31%	13.85%	12.55%	31.78%
1	19.98%	0.37%	20.54%	1.02%	9.16%	10.18%	38.76%

Table S3 Number of geometries validation classes using PM6-D3H4.

Method	*Mogul validation result*		
PM6-D3H4	superior		1352
PM6-D3H4	superior	side chain	166
PM6-D3H4	satisfactory		8501
PM6-D3H4	satisfactory	reasonable std	10347
PM6-D3H4	satisfactory	side chain	346
PM6-D3H4	awesome		4346
PM6-D3H4	awesome	side chain	28
		TOTAL	25086

Schema S1. Example of the input file for Mogul command line use.

```
MOGUL MOLECULE FILE 1SH.cif_mogul.mol2
MOGUL OUTPUT FILE 1SH.cif_validate.log
MOGUL EDIT BOND_TYPES GUESS UNKNOWN_3D
MOGUL EDIT BOND_TYPES STANDARDISE ALL ON
MOGUL EDIT HYDROGENS GENERATE NONE
MOGUL OUTPUT DISTRIBUTION ALL ON
CONFIG SEARCH ALL GENERALISATION ON
CONFIG SEARCH BOND MIN_OBSERVATIONS GENERALISED 15
CONFIG SEARCH ANGLE MIN_OBSERVATIONS GENERALISED 15
CONFIG SEARCH TORSION MIN_OBSERVATIONS GENERALISED 40
CONFIG SEARCH RING MIN_OBSERVATIONS GENERALISED 15
CONFIG SEARCH BOND MIN_RELEVANCE 0.75
CONFIG SEARCH ANGLE MIN_RELEVANCE 0.75
CONFIG SEARCH TORSION MIN_RELEVANCE 0.75
CONFIG SEARCH RING MIN_RELEVANCE 0.75
CONFIG SEARCH BOND SELECT BEST
CONFIG SEARCH ANGLE SELECT BEST
CONFIG SEARCH TORSION SELECT BEST
CONFIG SEARCH RING SELECT BEST
CONFIG SEARCH ALL FILTER RFACTOR 0.05
CONFIG SEARCH ALL FILTER EXCLUDE_ORGANOMETALLICS
CONFIG SEARCH ALL FILTER EXCLUDE_SOLVENTS
CONFIG OUTPUT INVALID_FRAGMENTS EXCLUDE
CONFIG CLASSIFICATION BOND    UNUSUAL z-score 2
CONFIG CLASSIFICATION ANGLE   UNUSUAL z-score 2
CONFIG CLASSIFICATION TORSION UNUSUAL local_density 5 WITHIN 10
CONFIG CLASSIFICATION RING    UNUSUAL local_density 5 WITHIN 10
CONFIG OUTPUT FORMAT TSV
CONFIG OUTPUT HEADER ON
CONFIG DISTRIBUTION BOND BIN_WIDTH 0.01
CONFIG DISTRIBUTION ANGLE BIN_WIDTH 2.0
BOND ALL
ANGLE ALL
TORSION ALL
RING ALL
```
